# Intravenous Terbutaline as Rescue Therapy in Severe Bronchiolitis: A Report of Two Cases

**DOI:** 10.12669/pjms.42.6.15590

**Published:** 2026-06

**Authors:** Wajiha Rizwan, Saba Urooj, Azher Abbas Shah, Adnan Tariq Bhutta

**Affiliations:** 1 Wajiha Rizwan, FCPS (Pediatrics), MHPE Associate Professor, Pediatric Medicine Department, University of Child Health Sciences, The Children’s Hospital Lahore, Lahore - Pakistan; 2 Saba Urooj, FCPS (Pediatrics), Senior Registrar, Pediatric Medicine Department, University of Child Health Sciences, The Children’s Hospital Lahore, Lahore - Pakistan; 3 Azher Abbas Shah, FCPS (Pediatrics) Professor, Pediatric Medicine Department, University of Child Health Sciences, The Children’s Hospital Lahore, Lahore - Pakistan; 4 Adnan Tariq Bhutta, MBBS, FAAP, FCCM, Professor of Clinical Pediatrics, Division Chief, Pediatric Critical Care Medicine, Indiana University School of Medicine and Riley Children’s Health

**Keywords:** β_2_-adrenergic agonists, Bronchiolitis, Infant, Respiratory syncytial virus, Terbutaline

## Abstract

Bronchiolitis is a lower respiratory tract infection most frequently caused by viral infections and is usually managed with supportive care. The role of bronchodilators particularly intravenous (IV) β_2_-agonists remain uncertain. Emerging evidence suggests that bronchiolitis comprises heterogeneous phenotypes, with a subset demonstrating bronchodilator responsiveness. We report two infants (ten and three months old) with RSV confirmed severe bronchiolitis who exhibited respiratory distress and hypoxemia despite maximal supportive therapy, including oxygen, continuous positive airway pressure (CPAP), corticosteroids and inhaled salbutamol. IV terbutaline was initiated as rescue therapy after failure of supportive treatment and titrated up to 0.4 µg/kg/min resulting in progressive improvement in clinical respiratory scores, resolution of wheeze, and normalization of oxygen saturation within 24-48 hours. No adverse effects were observed. These case reports highlight the potential role of IV terbutaline as a rescue therapy in selected infants with bronchiolitis associated bronchospasm and warrant further well designed larger prospective studies.

## INTRODUCTION

Bronchiolitis affects one third of children under two years of age.^1^ Respiratory syncytial virus (RSV) is most common causative agent associated with forty to ninety percent of bronchiolitis cases in children under five years of age.^2^ The presentation of bronchiolitis is usually mild but can even lead to respiratory failure and death especially in pre-term infants and those who have underlying immunodeficiency, chronic lung or heart disease.^1^,^2^ The underlying pathophysiology of bronchiolitis includes airway inflammation, edema, and mucus plugging that cause airflow limitation, wheeze and respiratory distress. Current management guidelines of National Institute for Health and Care Excellence (NICE) focus mainly on supportive care such as oxygen supplementation, hydration, and adequate nutrition, while routine use of bronchodilators, corticosteroids, or antibiotics is generally not advised.^3^

Even though the guidelines advise against using beta-2 agonists regularly, they are still widely used in real-world medical practice, with rates ranging from 18 to 90 percent reflecting uncertainty in identifying responders.^4^ Emerging evidence suggest that bronchiolitis represents a spectrum of disease with distinct phenotypes and endotypes, affected by age, atopic background, viral cause and underlying airway pathology.^4^,^5^ Recent research suggests that certain subtypes of bronchiolitis, especially involving older babies (>6 month) who have wheezing as the main symptom or show signs of allergies and particularly those demonstrating features of airway hyper reactivity, might get some help from bronchodilators use.^4^,^5^ A study including 995 children having bronchiolitis showed that using nebulized epinephrine helped lower the chance of being admitted to the hospital on the first day after an emergency visit. However, this effect didn’t last by day seven. So, it is suggested that epinephrine might be used in severe cases of bronchiolitis, but only under careful watch, and it should be stopped if there’s no sign of improvement in the child’s symptoms.^6^

The fact that responses to treatment vary and that benefits from current therapies don’t last highlights the need to look into other options, like IV terbutaline for severe bronchospasm when inhaled bronchodilator delivery to distal airway is limited.^7^ We report benefits of using IV terbutaline in two previously healthy full-term infants with severe bronchiolitis managed in high dependency unit of our pediatric department. They both had uneventful birth history and no family history of asthma or atopy. Written informed consent for the publication was given by the parents. Institutional approval was taken (No/818/UCHS-CH) as treatment was according to expert-consensus based protocols within the institution, based on previously observed clinical benefits.

## CASE-1 PRESENTATION

A 10-month-old female infant presented with complaints of fever and cough for three days and worsening respiratory distress for one day. At admission she was tachypneic, febrile and hypoxic (SpO2 88% in room air). Examination and lab investigation^*^ supported diagnosis of viral bronchiolitis (Table-I). Systemic corticosteroids (hydrocortisone), and inhaled salbutamol every six hours was started for wheeze which was initially thought to be caused by a possible bronchospastic issue. Despite maximal supportive management, respiratory distress worsened within initial six hours, with oxygen saturation remaining 91% on bubble CPAP (5 L/min at 5 cm H2O) and Clinical Respiratory Score^8^ of 10, indicating severe distress. IV terbutaline infusion was initiated (withholding inhaled salbutamol) at 0.1 µg/kg/min and titrated up to 0.4 µg/kg/min (increased 0.1 ug/kg/min based on clinical response). Following terbutaline initiation, at 6 hours CRS was seven, oxygen saturation normalized, and distress was improved. The CRS progressively improved and was five at 24 hours and there was only occasional wheeze, and the patient was that time shifted to low flow oxygen (3 L/min through nasal prong and tapered further while maintaining saturation above 94%). At 48 hours, wheeze settled, and patient was oxygen free with CRS 1. Terbutaline was tapered over the next 24 hours, no side effects were noted (ECG 12 hourly, serum potassium checked daily). Meanwhile inhaled salbutamol was continued every eight hours during this tapering and next day reduced to 12 hourly nebulization. The patient was asymptomatic and discharged on day five.

**Table-I:** Clinical Characteristics, Management, and Outcomes of the Two Infants with Severe Viral Bronchiolitis Treated with Intravenous Terbutaline.

Parameter	Case 1	Case 2
Age / Gender	10-month-old female	3-month-old female
Duration of illness before admission	Fever & cough: 3 days; respiratory distress: 1 day	Fever & cough: 4 days; respiratory distress: 1 day
Admission heart rate (beats/min)	126	138
Admission respiratory rate (breaths/min)	68	72
Admission temperature	101°F	101.5°F
Oxygen saturation on room air	88%	85%
Clinical examination	Marked subcostal and intercostal retractions, bilateral wheeze, reduced air entry with normal heart sounds and no audible murmur	Severe chest retractions, bilateral wheeze with occasional crepitations, decreased air entry with normal heart sounds and no audible murmur
Initial laboratory findings	WBC 13,200/µL (lymphocytes:59.2%,* granulocytes 37.3%); Hb 8.5 g/dL	WBC 7,000/µL (lymphocytes:58.8%,* granulocytes 36.6%), Hb 12.1g/dl
Blood culture	Negative	Negative
Viral PCR	Positive for RSV*	Positive for RSV*
Chest radiograph findings	Hyperinflation with prominent perihilar markings*	Bilateral perihilar infiltrates with hyperinflation; no focal consolidation*
Initial supportive management	CPAP oxygen, IV fluids, IV ceftriaxone, systemic corticosteroids, inhaled salbutamol	CPAP oxygen, IV fluids, IV ceftriaxone, systemic corticosteroids, inhaled salbutamol
Time to clinical deterioration	Within 6 hours	Within 4 hours
Oxygen saturation on CPAP at deterioration	91%	90%
Clinical Respiratory Score at deterioration	10 (severe)	11 (severe)
Terbutaline starting dose	0.1 µg/kg/min IV	0.2 µg/kg/min IV
Maximum terbutaline dose	0.4 µg/kg/min IV	0.4 µg/kg/min IV
Response to terbutaline	Wheeze resolved, CRS was 7 at 6 hours, 5 at 24 hours and 1 at 48 hours	Wheeze resolved, CRS was 8 at 6 hours, 5 at 24 hours at 0 at 48 hours
Duration of terbutaline therapy	72 hours (tapered over last 24 hours)	60 hours (tapered over last 24 hours)
Duration of oxygen therapy	48 hours after initiation of terbutaline	36 hours after initiation of terbutaline
Side Effects	No	No
Length of hospital stay	5 days	5days
Follow-up after 2 days of discharge	Asymptomatic	Asymptomatic

## CASE-2 PRESENTATION

A three-months-old female infant presented with complaints of fever and cough for four days and worsening respiratory distress for one day. At admission she was tachypneic, febrile and hypoxic (SpO2 85% on room air and 90% on bubble CPAP (6 L/min at 5 cm H2O). Patient was given hydrocortisone and inhaled salbutamol every six hours. Despite supportive therapy, clinical respiratory score^8^ was 11, prompting initiation of IV terbutaline at 0.2 µg/kg/min, increased to 0.4 µg/kg/min (withholding inhaled salbutamol) with oxygen saturation maintaining above 94% (increased 0. 1 µg /kg/min every 30 minutes). CRS improved gradually and was eight at six hours, five at 24 hours with marked clinical improvement. The wheeze settled after 24 hours, and oxygen was tapered to low flow oxygen via nasal prong (2L/min) and as baby started maintaining saturation >94% in room air so oxygen was stopped at 36 hours. Terbutaline was tapered over the following next 24 hours while continuing inhaled salbutamol eight hourly, CRS was 0 at 48 hours. The next day inhaled salbutamol was reduced to 12 hourly and held next day. No side effects of terbutaline were documented. The infant was discharged on day five.

## DISCUSSION

Bronchiolitis is a leading cause of hospital admission in infants and treatment is mainly supportive. Current NICE and AAP (American academy of Pediatrics) guidelines do not recommend routine use of steroids and inhaled bronchodilators because randomized control trials have not shown consistent benefits.^5^ Notably, the role of IV bronchodilators like terbutaline in bronchiolitis associated severe bronchospasm haven’t been systematically explored previously and hence its role in this context remains unknown.

A previous randomized controlled trial including 35 infants suffering from bronchiolitis showed no significant improvement in respiratory distress scores, oxygen saturation or respiratory rate during first two hours of terbutaline nebulization.^9^ In contrast, our reported cases demonstrate rapid improvement in these parameters after initiating IV terbutaline in refractory bronchiolitis cases within first six hours. However, it is known that some infants with bronchiolitis may exhibit a clinical pattern marked by severe wheezing, prolonged exhalation and possible severe bronchospasm^5^ like in our reported cases. In these situations, using inhaled bronchodilators might not be effective, as factors like rapid breathing, weak inhalation effort, and obstruction in the smaller airways can prevent the medication from reaching the affected areas properly. As a result, intravenous bronchodilators might be more dependable for delivering the medication systemically when there is a suspicion of severe bronchospasm.^7^

In our two reported infants with viral bronchiolitis, respiratory distress continued even after they received CPAP, oxygen, inhaled bronchodilators, corticosteroids, and other supportive treatments. They had wheezing, decreased breath sounds, and persistent hypoxemia, which led to the use of IV terbutaline as a last-resort treatment in our high dependency unit. The starting dose was slightly higher in second case due to higher CRS and lower oxygen saturation. While it’s not possible to confirm that terbutaline was the direct cause of clinical improvement of our reported case due to the other ongoing medications, yet as their condition improved shortly after starting the IV terbutaline suggesting temporal association. From a resource- limited setting that lack ventilated intensive care facilities, IV terbutaline might be thought about carefully as a possible treatment option for specific cases that don’t respond to standard care including high flow oxygenation and where bronchospasm is suspected, though more research is needed.

## CONCLUSION

Our experience suggests the potential role of IV terbutaline as a short-term rescue therapy in severe bronchiolitis; however, well designed larger prospective studies are required to confirm its efficacy and safety before broader adoption.

### Author’s Contribution:

**WR:** Conceived, designed and did manuscript writing.

**SU:** Did data collection and manuscript writing.

**AAS:** Designed, did critical review of manuscript and responsible for integrity of research.

**ATB:** Did critical review and final approval of manuscript.
